# Observational evidence for enhanced magnetic activity of superflare stars

**DOI:** 10.1038/ncomms11058

**Published:** 2016-03-24

**Authors:** Christoffer Karoff, Mads Faurschou Knudsen, Peter De Cat, Alfio Bonanno, Alexandra Fogtmann-Schulz, Jianning Fu, Antonio Frasca, Fadil Inceoglu, Jesper Olsen, Yong Zhang, Yonghui Hou, Yuefei Wang, Jianrong Shi, Wei Zhang

**Affiliations:** 1 Department of Geoscience, Aarhus University, Høegh-Guldbergs Gade 2, 8000 Aarhus C, Denmark; 2 Stellar Astrophysics Centre, Department of Physics and Astronomy, Aarhus University, Ny Munkegade 120, 8000 Aarhus C, Denmark; 3 Royal Observatory of Belgium, Ringlaan 3, B-1180 Brussel, Belgium; 4 INAF-Osservatorio Astrofisico di Catania, via S.Sofia 78, 95123 Catania, Italy; 5 Department of Astronomy, Beijing Normal University, 19 Avenue Xinjiekouwai, Beijing 100875, China; 6 AMS, 14C Dating Centre, Department of Physics, Aarhus University, Ny Munkegade 120, 8000 Aarhus C, Denmark; 7 Nanjing Institute of Astronomical Optics and Technology, National Astronomical Observatories, Chinese Academy of Sciences, Nanjing 210042, China; 8 Key Laboratory of Optical Astronomy, National Astronomical Observatories, Chinese Academy of Sciences, Beijing 100012, China

## Abstract

Superflares are large explosive events on stellar surfaces one to six orders-of-magnitude larger than the largest flares observed on the Sun throughout the space age. Due to the huge amount of energy released in these superflares, it has been speculated if the underlying mechanism is the same as for solar flares, which are caused by magnetic reconnection in the solar corona. Here, we analyse observations made with the LAMOST telescope of 5,648 solar-like stars, including 48 superflare stars. These observations show that superflare stars are generally characterized by larger chromospheric emissions than other stars, including the Sun. However, superflare stars with activity levels lower than, or comparable to, the Sun do exist, suggesting that solar flares and superflares most likely share the same origin. The very large ensemble of solar-like stars included in this study enables detailed and robust estimates of the relation between chromospheric activity and the occurrence of superflares.

## Abstract (web summary)

Superflares are large explosive events on stellar surfaces, much larger than solar flares, but it remains unclear whether they share the same origin. Here, the authors analysed 48 superflare stars and determine the relation between their chromospheric activity and the occurrence of superflares.

## Graphical abstract

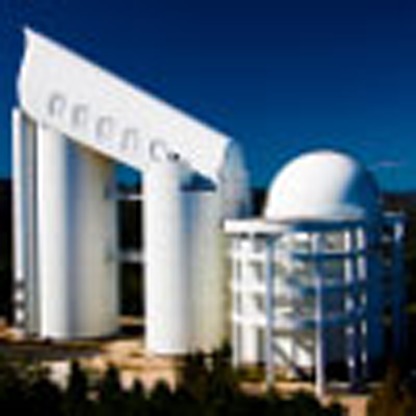

The largest known solar flare was the Carrington event in AD 1859 (refs 1, 2). This flare and the associated coronal mass ejection were so large that they caused world-wide auroras and allowed telegraphs to operate on the currents induced by the accompanying geomagnetic storm^3^. As we have no X-ray measurements of the Carrington event, it is not clear how large it was compared with the largest flares observed during the space age, which are classified according to their peak X-ray flux. Estimates based on magnetometer traces predict that the Carrington event, with a total energy of up to 5·10^32^ erg, was likely larger than any solar flare observed in the space age^4^. On the other hand, it is clear that the hazard caused by the Carrington event was minimal compared with that potentially posed by, for example, a 2·10^34^ erg superflare. Fifteen years ago, Schaefer *et al*.^5^ identified nine so-called superflares, defined as flares with energies ranging from 10^33^ to 10^38^ erg, on nine ordinary solar-type stars. We call these stars superflare stars.

Superflares on solar-like stars may arise from at least three different mechanisms, apart from coronal magnetic reconnection: (i) star–star interactions, as is the case for RS CVn systems, where a close binary companion tidally spins up an F or G main-sequence star. It has been suggested that this tidal interaction can be accompanied by large co-rotating flux tubes that temporarily may connect the two stars and thus cause superflares when the interconnecting field lines subsequently disrupt^6^; (ii) star–disk interactions, where the dipole magnetic field of the central star is connected to the co-rotating disk. The twist imposed on the dipole magnetic field can thus lead to disruption and superflares^7^; (iii) star–planet interactions, which can take place in two different ways, either similar to the star–star mechanism through disruption of interconnecting field lines^8^,^9^ or through tidal interaction between the star and the planet, which can lead to enhanced dynamo action^10^ and thus greater magnetic reconnection events. One of the main differences between the star–star and the star–planet mechanisms is that where RS CVn-type stars generally have activity levels many times higher than those found in even the most active single solar-like stars^11^, stars hosting Jupiter-like planets have activity levels comparable to average solar-like stars^12^. All three mechanisms were analysed theoretically by Shibata *et al*.^13^ along with the possibility for a Sun-like star to generate a superflare through coronal magnetic reconnection. It was found that it would take the Sun 40 years to generate a sunspot large enough and with a magnetic field strong enough to produce a 10^35^ erg superflare. Although Shibata *et al*.^13^ conclude that the coronal magnetic reconnection hypothesis is the most like scenario, they were not able to rule out other scenarios, such as enhanced dynamo action through tidal interactions.

Using 120 days of high-precision, high-cadence photometric observations from NASA's Kepler mission^14^, Maehara *et al*.^15^ identified 365 superflares on 148 solar-type stars. This study was updated by Shibayama *et al*.^16^ using 550 days of observations to include 1,547 superflares on 279 G-type stars. The nature of these has been investigated by a number of authors who analysed periodic brightness modulations in their Kepler light curves^15^,^16^,^17^,^18^,^19^. All these studies show that fast rotating stars are more likely to host superflares, but in general, the measured rotation periods have to be handled with great caution as: it has been shown using simulations that Kepler observations cannot be used to extract rotation periods for stars where the spot life-time is comparable to or shorter than the rotation period, as is the case for the Sun^20^; the Kepler light curves are heavily affected by artefacts on time scales longer than 20 days^21^; a large fraction of the apparently rapid rotating (a few days) stars are likely ellipsoidal binaries^22^,^23^.

Instead of looking at the rotation rates, the activity levels can be measured as emission in the Ca II H and K spectral lines at 396.8 and 393.4 nm, respectively. The intensity of the emission scales with the amount of non-thermal heating in the chromosphere, making these lines a useful proxy for the strength of, and fractional area covered by, magnetic fields. This was first suggested by Eberhard and Schwarzschild^24^ and has subsequently been used extensively to measure stellar activity since Olin Wilson started regular observations at the Mount Wilson Observatory^25^. The emission in the Ca II H and K lines is measured by the canonical S index, which intensive research over the last 4 decades has shown is related to magnetic activity^26^,^27^.

Stellar activity can also be measured using, for example, the Ca II infrared triplet or the Hα line^28^,^29^,^30^,^31^. Notsu *et al*.^31^ used the Subaru telescope to measure activity levels, using the Ca II line at 8,542 Å, of 27 superflare stars, which all show activity levels larger than the Sun. A strong correlation was observed between the photometric modulation of the Kepler light curves over the rotation period and the measured activity levels. It is, however, not clear from these observations if all superflare stars have activity levels higher than the Sun, as the Ca II line at 8,542 Å is insensitive to activity at activity levels lower than or compared with the activity level of the Sun^31^,^32^.

Here, we use observations from the Large Sky Area Multi-Object Fibre Spectroscopic Telescope (LAMOST, also called the Guoshoujing Telescope at Xinglong observatory, China)^33^ to show that superflare stars are generally characterized by larger chromospheric emissions than other stars, including the Sun. However, superflare stars with activity levels lower than, or comparable to, the Sun do exist, suggesting that solar flares and superflares most likely share the same origin. The very large ensemble of solar-like stars included in this study enables detailed and robust estimates of the relation between chromospheric activity and the occurrence of superflares.

## Results

### Superflare stars are characterized by enhanced activity

**Figure 1:**
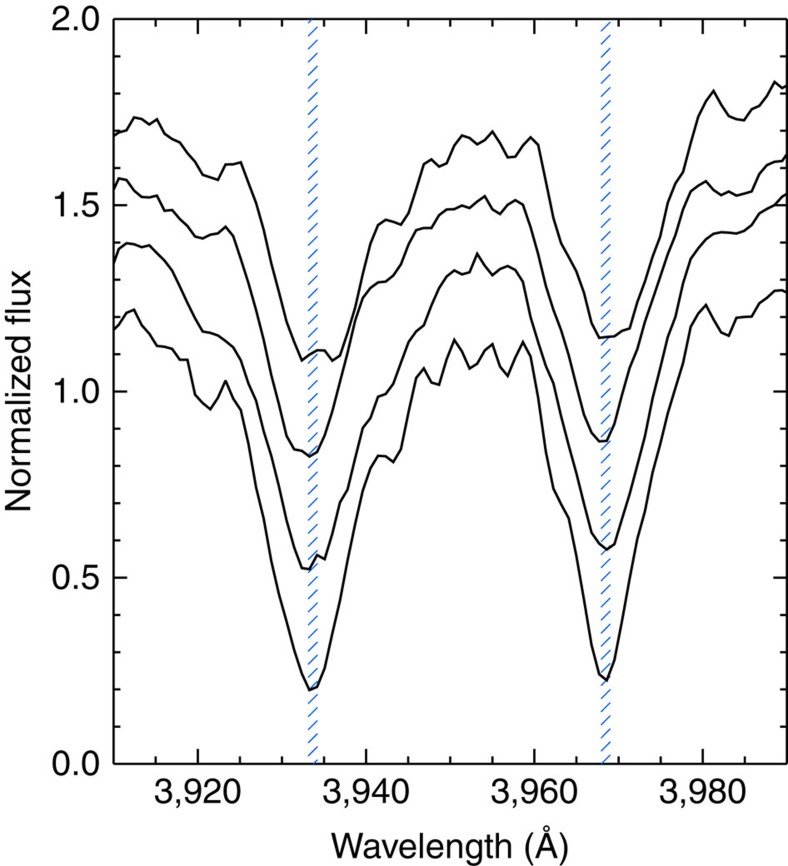
Examples of spectra showing different chromospheric emission levels in four solar-like stars. The spectra show the Ca II H and K absorption lines at 396.8 and 393.4 nm, respectively. The four stars shown here are, bottom to top, KIC 8493735, KIC 9025370, KIC 8552540 and KIC 8396230. The spectra for these four stars have been normalized to one in the spectral range shown, but for clarity we have applied an offset of 0.2 between each spectrum. The measured S index for these four stars are: 0.15, 0.23, 0.30 and 0.34, respectively. The difference in S index can be seen as the increase in emission in the core of the absorption lines. The blue shaded regions show the wavelength bands used to measure the S index.

**Figure 2:**
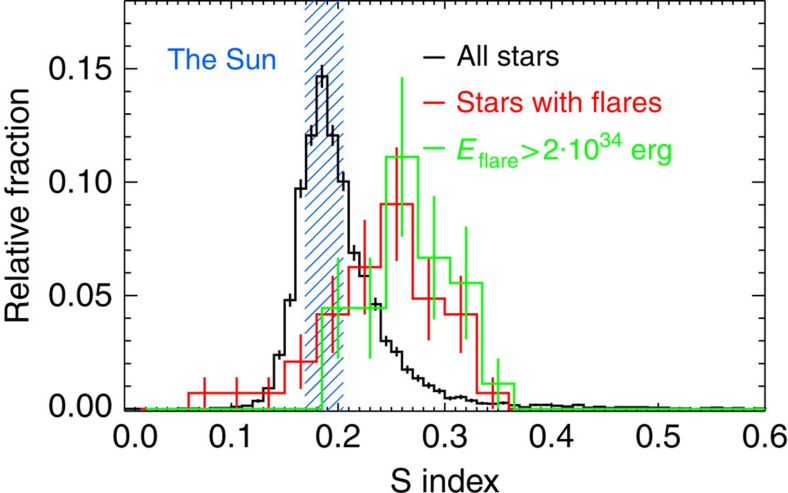
Histograms of the activity distribution of solar-like stars. (main-sequence stars with effective temperatures between 5,100 and 6,000 K) in black compared with those of these stars that show superflares in their Kepler light-curve in the analysis of Shibayama *et al*.^16^ in red. The blue shaded region marks the range of the S index of the Sun between solar cycle minima and maxima^34^. The two distributions are different at a 6σ level, clearly showing that the superflare stars generally have higher activity levels than average solar-like stars. The green curve shows the distribution of superflare stars with total energies larger than 2·10^34^ erg in the analysis of Shibayama *et al*.^16^ It is seen that this distribution is shifted to even higher activity levels. The error bars are based on the number of stars in each bin, assuming Poisson statistics. The uncertainties on the measured S indicies are smaller than 0.03 (see Supplementary Fig. 2 for details). The error bars in the figure represent a single standard deviation.

Using observations from LAMOST, we have measured the S index of 5,648 main-sequence, solar-like stars with effective surface temperatures between 5,100 and 6,000 K in the field of view of the Kepler mission (Methods). Figure 1 shows four examples of spectra of the Ca II H and K lines for solar-like stars with different activity levels. In all, 48 of these 5,648 stars have been categorized as superflare stars by Shibayama *et al*.^16^. We compare the distribution of the measured activity levels of all the 5,648 solar-like stars with the distribution of the 48 stars that show superflares (Fig. 2). The two distributions are clearly different, with the activity levels of the superflare stars shifted to higher values. A Kolmogorov–Smirnov test shows that the two distributions are different at the 99.999999983% significance level, comparable to 6*σ*. This result shows that superflare stars generally exhibit stronger activity levels than other solar-like stars, indicating that the superflare stars have larger dynamo actions and thus that the superflares may be caused by a mechanism similar to the one generating solar flares. This is also supported by a bootstrap test, which shows that the mean S index of the superflare stars falls within the top 0.002% of the distribution of mean S indices when 48 stars are repeatedly selected at random from the 5,648 main-sequence stars (Methods).

If the activity distribution of the superflare stars is compared with the activity level of the Sun, which has an S index that varies between 0.169 and 0.205 from minimum to maximum of the 11-year solar cycle^34^, we observe that superflare stars generally have activity levels larger than what is observed on the Sun. In all, 42 out of 48 superflare stars have activity levels larger than 0.169, whereas 36 out of 48 superflare stars have activity levels larger than 0.205. If we only consider superflares with total energies larger than 2·10^34^ erg, we observe that 30 out of 30 stars have activity levels larger than 0.169, whereas 26 out of 30 have activity levels larger than 0.205 (Methods). The distribution of stars hosting superflares with total energies larger than 2·10^34^ erg is shifted to higher activity levels, and none of these superflare stars display activity levels significantly lower than the Sun (Fig. 2). However, it is noteworthy that 12 of 48 of superflare stars and 4 of 30 superflare stars with superflares above 2·10^34^ erg have chromospheric emission levels comparable to the Sun.

### Comparison to the Subaru observations

**Figure 3:**
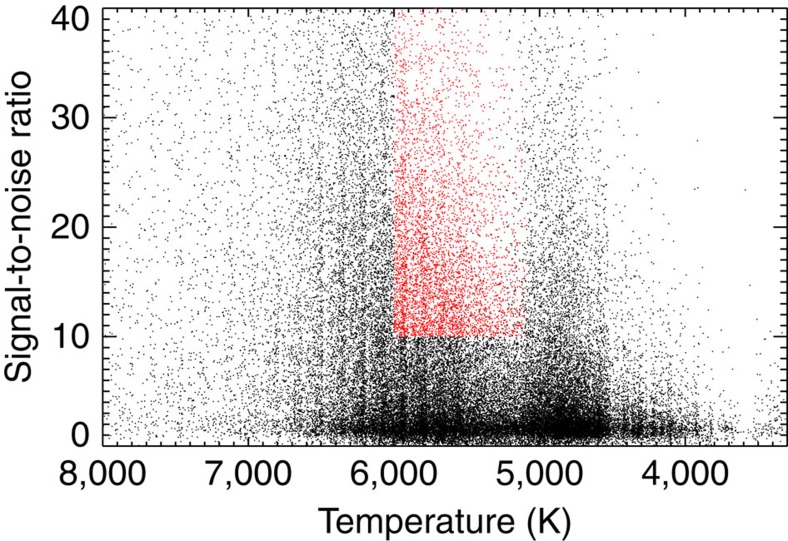
Relationship between the signal-to-noise ratio in the blue part of the spectra and effective temperature. The effective temperatures are from Brown *et al*.^58^ and the signal-to-noise ratio calculated as described by Luo *et al*.^35^ The 5,648 main-sequence stars with signal-to-noise ratio higher than 10, which we analyse in this study, are marked with red. A clear separation between main-sequence (around 6,000 K) and evolved stars (around 5,000 K) can be seen as a function of effective temperature.

**Figure 4:**
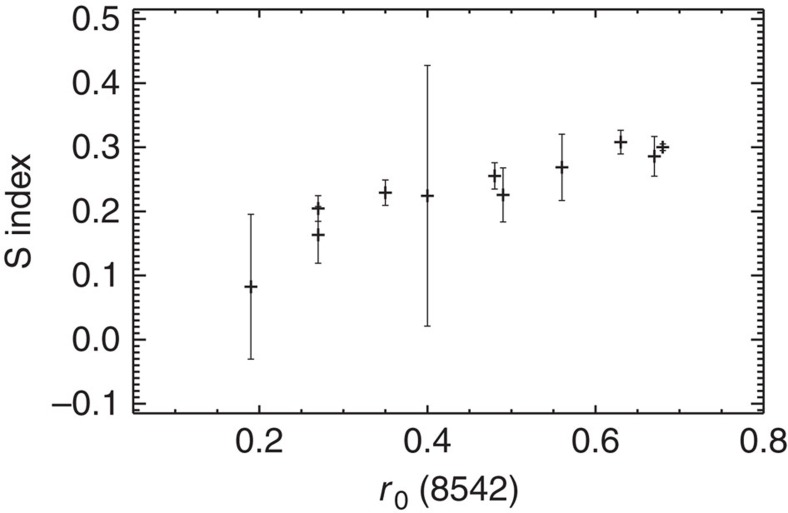
Relation between residual flux in the core of the infrared Ca II 8542 line and the S index for 11 stars that have been observed with both the Subaru and LAMOST telescope. A clear correlation is seen between the two activity indicators. The clear correlation between the measured activities indicates that the S index we obtain with LAMOST observations is a reliable measure of chromospheric emission. The error bars are calculated using the relation in Supplementary Fig. 2. The error bars in the figure represent a single standard deviation.

In this study, we only analyse stars with a signal-to-noise ratio higher than 10 in the blue part of the spectrum, as spectra with lower signal-to-noise ratio do not allow the S index to be measured with the required accuracy. The signal-to-noise ratio is measured as described in Luo *et al*.^35^ and shown in Fig. 3. The risk associated with using signal-to-noise ratios down to 10 is that the activity measurements may become unreliable. To test this, we have compared our activity measurements (S index) with activity measurements based on the residual flux in the core of the infrared Ca II 8,542 line (*r*_0_) by Notsu *et al*.^31^ (Fig. 4). The clear correlation between the measured activities indicates that the S index we measure with the LAMOST observations is a reliable measure of chromospheric emission. We also tested the reliability of the measured activity levels by only analysing spectra with a signal-to-noise ratio higher than 30. Although this lowered the number of analysed stars dramatically, it did not change any of the conclusions.

Notsu *et al*.^31^ also calculated the magnetic flux *<fB>* using the residual flux in the core of the infrared Ca II line. Using the formulation of Schrijver *et al*.^27^, we also calculate the magnetic flux based on our measured S index (Methods). These fluxes agree with those of Notsu *et al*.^31^ within the uncertainties, although the uncertainties associated with the estimated magnetic fluxes are relatively large in both cases (Methods). The uncertainties are in both cases calculated based on the transformation to magnetic flux *<fB>* using solar data. It is therefore not clear how the accuracy of the two sets of activity measurements compare with one another. On the one hand, the Subaru spectra have higher resolution and higher signal-to-noise ratio than the LAMOST spectra, but, on the other hand, the S index is a better activity indicator than the residual flux in the core of the infrared Ca II line. The main difference between the Subaru and the LAMOST observations is the much larger (5,648) sample size of the LAMOST observations, which allow us to conclude that superflare stars are generally characterized by higher activity levels than other solar-like stars.

### Comparison to star spot coverage

**Figure 5:**
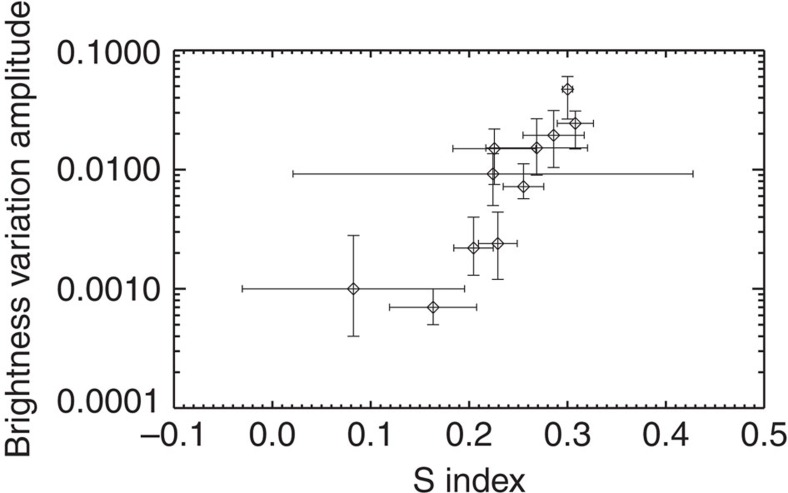
Relation between the S index and the stellar brightness variation. The brightness variation amplitude measurements are from Notsu *et al*.^31^ for the same 11 stars as in Fig. 4. A strong correlation is seen, which agrees well with the strong correlation seen between the residual flux in the core of the infrared Ca II 8542 line and the brightness variation amplitude by Notsu *et al*.^31^ The error bars are calculated using the relation in Supplementary Fig. 2. The error bars in the figure represent a single standard deviation.

**Figure 6:**
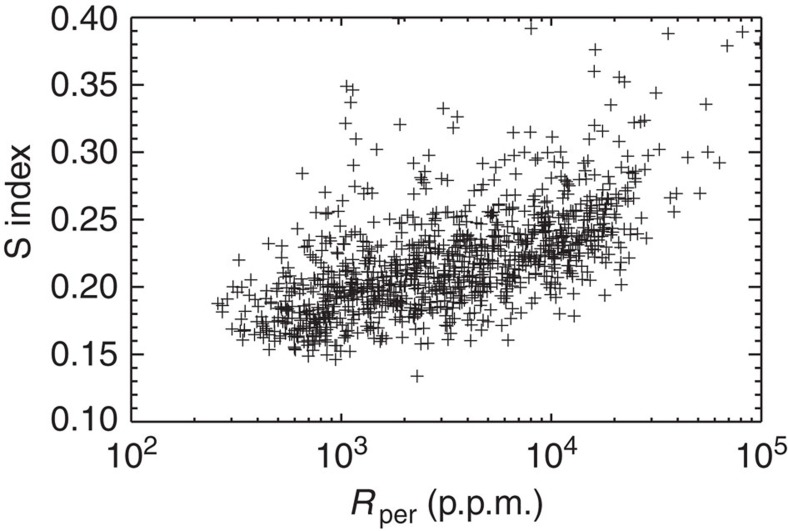
Relation between the periodic photometric variability amplitude and the S index. The periodic photometric variability amplitudes are from McQuillan *et al*.^36^ for 1,400 stars that also have LAMOST spectra that allow reliable measurements of the chromospheric emission. Indication of a correlation is seen down to periodic photometric variability amplitudes around 1,000 p.p.m., which is the level of the amplitudes seen in the Sun. Below 1,000 p.p.m. no clear indication of a correlation is seen, indicating that the relation between spot coverage and chromospheric emission breaks down when the magnetic activity is low.

Chromospheric emission, as measured with the S index, is one manifestation of stellar magnetic fields. Another manifestation is star spots. Notsu *et al*.^31^ compared the so-called mean stellar brightness variation with the residual flux in the core of the infrared Ca II line. The stellar brightness variation is calculated as the difference between the maximum and minimum of the stellar flux measured with Kepler within a given 3-months period (after outlier removal)^31^. The comparison shows a strong correlation between these two quantities. In Fig. 5, we thus compare our measured chromospheric emission with the stellar brightness variation measured by Notsu *et al*.^31^ Again, we see a strong correlation for the 11 stars that have been observed with both the Subaru and LAMOST telescopes. Unfortunately, 9 out of these 11 stars show both larger chromospheric emission, residual flux in the core of the infrared Ca II line and stellar brightness variation than the Sun. In order to test the relation between chromospheric emission and star spot coverage over a larger parameter range, we compare the measured S indices with the periodic photometric variability amplitude (*R*_per_) measured by McQuillan *et al*.^36^ (Fig. 6). The periodic photometric variability amplitude is calculated as the range between the 5th and 95th percentile of the normalized flux^36^. Indications of a correlation between the S index and the periodic photometric variability amplitude are observed down to periodic photometric variability amplitudes around 1,000 p.p.m. (Fig. 6), which is the level of the amplitudes observed for the Sun. Below 1,000 p.p.m., no clear indications of a correlation is seen, indicating that the relation between spot coverage and chromospheric emission breaks down when the magnetic activity is low. The comparison between the measured chromospheric emission and the photometric variability in both the studies by Notsu *et al*.^31^ and McQuillan *et al*.^36^ also indicate that the S index we measure with LAMOST is a reliable measure of chromospheric emission.

## Discussion

Based on activity measurements of 5,648 solar-like stars, including 48 superflare stars, we show that superflare stars are generally characterized by higher activity levels than other stars, including the Sun. However, superflare stars with activity levels lower than, or comparable to, the Sun do exist, but none of the stars hosting the largest superflares (>2·10^34^ erg) show activity levels lower than the Sun. As discussed above, superflares on solar-like stars may result from at least three different mechanisms apart from coronal magnetic reconnection: (i) star–star interactions, (ii) star–disk interactions and (iii) star–planet interactions. Our activity measurements show that superflare stars generally have S indices between 0.15 and 0.35. Although this is higher than solar-like stars in general and higher than the Sun, it is lower than typical RS CVn-type stars, which usually have S indices higher than 1.00 (ref. 11). It is therefore unlikely that a large fraction of the superflares can be explained by RS CVn-type star–star interaction.

Although we cannot evaluate if the star–disk and the star–planet hypotheses may be correct, it is likely that the magnetic field of the superflare stars should be significantly larger than what we observe on the Sun for these mechanisms to operate^8^. The fact that the lower part of the distribution of the measured activity of the superflare stars overlap with the range observed for the Sun indicates that these mechanisms most likely are not the main cause of superflares. Also, given the transit probability for a hot Jupiter in a 4-day circular orbit^37^ and because none of the superflare stars are known to host hot Jupiters, the star–planet mechanism is unlikely to be the main mechanism responsible for superflares^15^.

Our study provides observational (although non-exclusive) support for the coronal magnetic reconnection hypothesis, as we show that superflare stars generally exhibit higher chromospheric emissions than the Sun and other solar-like stars, although there is an overlap between the two distributions. The coronal magnetic reconnection hypothesis can explain the observations via the notion that superflares and solar flares share the same origin and that the two activity distributions therefore are within similar range, but that superflares mainly take place on stars with activity levels larger than the Sun. If the observations had shown two different and non-overlapping ranges of activity levels in superflare (red curve) and non-superflare stars (black curve), for example, centred on S indices around 1.00 and 0.18, respectively, it would have favoured the star–star, star–disk or star–planet mechanisms, but this is not the case.

**Figure 7:**
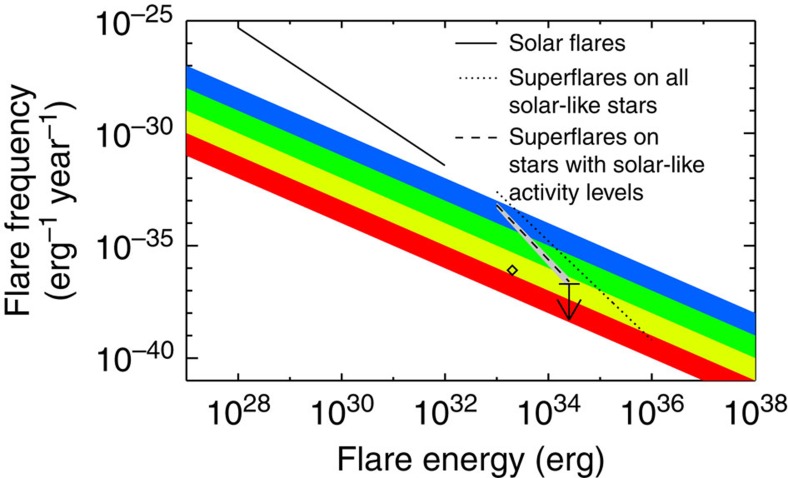
Comparison of occurrence rates of flares on the Sun and of superflares on solar-like stars. The solid line shows the power-law distribution of solar flares^64^, which is compared with the power-law distribution of superflares on the 279 G-type main-sequence stars found by Shibayama *et al*.^16^ (dotted line) and the power-law distribution of those of these stars that have activity levels lower than the Sun at solar cycle maximum (dashed line, Methods). The last power-law assumes that the Sun's chromospheric emission does not change beyond what have been observed so far. The grey region marks the 1*σ* error range. The diamond marks the probability of an AD 775 or AD 993 event, assuming that such an event takes place every 620 y. We have extended the power-law down to energies of 10^35^ erg, although the observations suffer from detection limit effects below energies of 5·10^34^ erg. The extension of the power-law to energies below 5 × 10^34^ erg is, however, supported by Maehara *et al*.^68^ The coloured regions mark where one flare per year (blue), decade (green), century (yellow) or millennium (red) is expected.

The observations also make it possible to renew the evaluation of the frequency of solar superflares (Fig. 7). Here, we have compared the frequency of solar flares with the frequency of superflares on solar-like stars as a function of the flare energy (Methods). If only the superflare stars with activity levels comparable to or smaller than the Sun are considered, then the frequency of 2·10^34^ erg superflares is reduced with almost an order-of-magnitude compared with solar-like stars in general.

This result confirms the result by Shibayama *et al*.^16^, who found that 1,150 out of 1,547 superflares occurred on fast rotating stars. Combined, the two results indicate that although superflares on solar-like stars with Sun-like rotation and chromospheric emission are an order-of-magnitude less likely than superflares on solar-like stars in general, they still occur. We do, however, have to be careful with the definition of Sun-like stars here. Shibata *et al*.^13^ suggested that a sunspot with a radius around 30% of the solar radius would be needed in order for the Sun to produce a superflare with an energy above 10^35^ erg. Although such a large spot has never be observed on the Sun, it is clear that the Sun would likely fall outside our definition of Sun-like, if such a large spot was observed, as such a large spot would likely result in large chromospheric emission.

The downward adjustment of the flare frequency based on the activity measurements leads to a better agreement between astrophysical activity observations of superflare stars and the frequency of solar flares recorded in geological archives (see Fig. 7 for details). Geological archives, in particular cosmogenic nuclides (^10^Be and ^14^C) in ice cores and tree rings, can be used to evaluate the flare frequency through so-called solar particle events, where protons are accelerated in connection with large solar flares to energies sufficiently high to produce cosmogenic nuclides when they reach the Earth's atmosphere (see Schrijver *et al*.^38^ for a recent review).

The raw flare frequency estimated from the Kepler data, calculated without weighting for activity, seems to roughly follow the power-law distribution of solar flares^16^,^17^,^39^. To some extent, this contradicts results from a number of geological archives, which indicate a break, or roll-over, in the distribution of solar particle events at energies around 10^33^ erg (refs 39, 40, 41, 42). The discussion of this roll-over effect has received renewed attention with recent discoveries based on studies of ^14^C in Japanese tree rings, indicating that the Sun hosted a superflare with an energy larger than 10^33^ erg in AD 775 (refs 43, 44, 45, 46, 47, 48). The solar origin of the AD 775 event was recently questioned due to the lack of simultaneous aurora observations^49^, but evidence from a new study based on multi-radionuclides suggest a solar origin^50^. A similar event might have taken place in AD 993 (ref. 51). In Fig. 7, the AD 775 and the AD 993 events are shown with a diamond, assuming that such events take place every 620 years. The upper-limit flare frequency weighted for activity lies over the AD 775 and AD 993 events. This does not contradict the roll-over scenario, but it does not place tight constraints on the occurrence of such events either. The indications of a roll-over effect in the flare frequency weighted for magnetic activity is also in agreement with recent theoretical estimates, which predict that sunspot groups larger than historically reported would be needed in order for the Sun to produce a superflare with an energy larger than ∼6·10^33^ erg (ref. 52).

## Methods

### The observations

The magnetic activity measurements from LAMOST enable the first direct comparisons of magnetic activity levels in superflare stars to the activity levels of solar-like stars in general. This is only possible because of the unique combination of a large aperture, large field-of-view and multi-object fibre spectroscopy.

### Calculation of the S index

The LAMOST-Kepler project is described by De Cat *et al*.^53^ For the present study, we analysed 71,733 low-resolution spectra (*R*∼1,800). These spectra were reduced as described in Luo *et al*.^35^ This reduction included standard bias and dark-frame subtraction, flat-field correction and extraction of a one-dimensional spectrum. All spectra were cross-correlated with a solar spectrum to place the observed spectra on a reference wavelength grid with velocities zeroed.

The emission in the Ca II H and K lines is measured by the canonical S index defined as^54^:


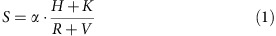


where *H* and *K* are the recorded counts in a 1.09 Å full-width at half-maximum triangular bandpasses centred on the H and K lines at 396.8 and 393.4 nm, respectively. *V* and *R* are two 20 Å wide reference bandpasses centred on 390.1 and 400.1 nm. *α* is a normalization constant.

As our observations are done with a spectrograph and not with a spectrophotometer, it is convenient to rewrite S as a function of mean flux per wavelength interval 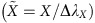:


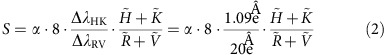


The factor of 8 comes from the fact that the Mt Wilson spectrophotometer used a rapidly rotating slit mask that exposed the H and K channels eight times longer than the reference channels. The value of *α*=1.8 is obtained from Hall, Lockwood and Skiff^55^. Other values of *α* can be found in the literature and these values are sometimes obtained by comparing S indices for the same stars measured with different instruments. Unfortunately, we have not been able to find other measurements of S indices for any of the Kepler stars observed by LAMOST. The main reason for this is likely that most surveys, especially on the northern hemisphere, focus on relatively bright stars, which saturate the LAMOST charge-coupled devices (CCDs)^53^. Instead, we have compared the distributions of the S indices from the LAMOST observations with the distribution of the S indices from Isaacson and Fischer^56^ (Supplementary Fig. 1). Here, it is seen that although the LAMOST observations do not see as many high-active stars with an S index above 0.5, the two distributions are identical around 0.20. If we only consider main-sequence stars with effective temperatures between 5,100 and 6,000 K and S indices between 0.1 and 0.3, the mean value of the LAMOST observations is 0.192±0.03 compared with 0.192±0.04 for the Isaacson and Fischer sample. This is confirmed by a Kolmogorov–Smirnov test, which shows that the two distributions are identical between 0.1 and 0.3 at the 93% significance level. For the stars in the Isaacson and Fischer^56^ sample, we define main-sequence stars as stars with absolute visual magnitude within 1 magnitude of the main sequence. The effective temperatures are calculated from the B-V colour index using the formulation by Alonso *et al*.^57^ The ensemble thus consists of 60 main-sequence stars with effective temperatures between 5,100 and 6,000 K and with S indices between 0.1 an 0.3. Based on this analysis, we conclude that *α*=1.8 is an appropriated choice.

The uncertainties associated with the S indices is found by comparing the standard deviation of different measurements of the S index for 16,900 main-sequence stars with effective temperatures below 6,200 K that are located in more than one LAMOST field^53^. In Supplementary Fig. 2, we show the standard deviation as a function of the mean signal-to-noise ratio in the blue part of the spectrum of these stars. We can use this relation to calculate the uncertainties of the measurements of the S indices as a function of the signal-to-noise ratio in the blue part of the spectrum as: 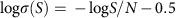. The calculation was carried out using *χ*^2^ minimization, yelling a reduced *χ*^2^ of 0.45. In the calculation, we assume that the measurements are not independent measurements of the same quantity, in which case we should have used the uncertainty of the mean value of the S indices as the uncertainty instead of the standard deviation of the different measurements of the S indices. This is done to achieve conservative estimates of the uncertainty and because we expect that the intrinsic chromospheric emission of the stars change between observations. This implies that the uncertainty we report also includes a stellar variability component. We do, however, note that the scatter is large in Supplementary Fig. 2 and that the uncertainties therefore should be handled with caution.

For each star, we made a cross-identification to the Kepler Input Catalog^58^ (KIC) and restricted the analysis to stars on the main sequence with effective temperatures between 5,100 and 6,000 K (the same range as used by Shibayama *et al*.^16^). The main sequence was defined by the green line in Supplementary Fig. 3. The measured S indices for the superflare stars are given in Supplementary Table 1 together with the effective temperatures and surface gravity.

Huber *et al*.^59^ collected fundamental stellar parameters for stars in the KIC from, for example, spectroscopic studies, binaries and planet studies and asteroseismology. Unfortunately, none of the stars analysed in our study were identified in any of these studies. Huber *et al*.^59^ also updated the fundamental parameters for 196,468 stars in the Kepler FOV using isochrones fitted to the collected fundamental stellar parameters. All the stars in our study thus have updated parameters by Huber *et al*.^59^ We show the updated values of the effective temperatures and surface gravity in Supplementary Fig. 4. Although both the effective temperature and surface gravity do change for some stars, it does not change any of the conclusions in our study and we have thus chosen to use the KIC values, as Huber *et al*.^59^ explicitly stress that their updated values are only accurate for large ensembles and not suitable for scientific analysis on a star-by-star basis. Nevertheless, we want to note that KIC 11197517 and KIC 11241343 could have evolved off the main-sequence, based on the updated surface gravities from Huber *et al*.^59^ Asteroseismic studies have previously shown that superflares are not uncommon on stars that have evolved off the main-sequence^60^. The updated effective temperatures imply that 13 out of the 48 superflare stars fall outside the 5,100–6,000 K effective temperature range when using the effective temperatures from Huber *et al*.^59^ In Supplementary Fig. 5, we thus show how Fig. 2, showing the histogram of the activity distributions, would look if the effective temperature and surface gravity values were taken from Huber *et al*.^59^ It is clear from this figure that this would not change the conclusions.

### Chromospheric flux

The S index is known to be colour dependent^61^. Also, the S index is a purely empirical quantity and it may be advantageous to calculate the chromospheric flux instead. The chromospheric flux can be calculated from the S index and some colour information like B-V and or effective temperature. The precision on the calculated chromospheric flux therefore depends on the precision of the colour information. We use the effective temperatures from KIC to calculate the B-V colour index using the formulation by Alonso *et al*.^57^

The chromospheric flux is calculated as 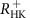 and 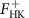, using the formulation by Mittag *et al*.^62^ Here, the chromospheric emission, the S index, is converted into physical units and corrected for both photospheric flux and a so-called basal flux, in order to calculate a pure activity-related quantity. The uncertainties are found by combining the uncertainty of the S index with 100 K uncertainty on the effective temperature from KIC. We also show the same histogram of the distribution of 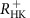 and 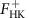 in solar-like stars in general, in superflare stars and in stars with superflares with total energies larger than 2·10^34^ erg in Supplementary Figs 6 and 7, as we showed for the S index in Fig. 2. These two figures clearly confirm the results based on the S index, that is, superflare stars are generally characterized by higher activity levels than other stars, but that superflare stars with activity levels lower than, or comparable to, other solar-like stars do exist. Supplementary Figs 6 and 7 do, however, indicate that the noise in the chromospheric flux measurements is larger than in the S index. This is likely due to both noise in the effective temperature from KIC and in the conversion from effective temperature to the B-V colour index. A test showed that using the effective temperatures from Huber *et al*.^59^ did not change the results.

Supplementary Data 1 contains S, 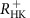 and 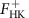, as well as uncertainties, for all the 5,648 main-sequence solar-like stars.

### Correlation between stellar activity and flare energy

In Supplementary Fig. 8, we show the relation between the total bolometric flare energy and stellar activity. Except for the absence of any high-energy flares with weak stellar activity, no clear correlation is seen. This is not unexpected, as stars with strong stellar activity are expected to host both medium and larger superflares.

The Kepler photometry of the three stars with S index below 0.13 (KIC 2850378, KIC 11197517 and KIC 11241343) could be contaminated by background stars. If this is the case, it would explain both the low value of the total bolometric flare energy (as the absolute luminosity of the superflare stars and thus the total bolometric flare energy would be larger) and the S index (as the light entering the fibre would likely be contaminated and the observed emission in the H and K lines would therefore be relatively small). On the other hand, we do not have any prime indications that these stars are contaminated by background stars. Also, the spectra did not show any signs of binarity or artefacts, so although these three superflare stars with low S index are hard to explain with any of the proposed scenarios, we do not find any observational bias that can remove them from the sample population. The low activity of these three stars could indicate that these stars are in a grand activity minimum, similar to what the Sun went through during the Maunder Minimum^63^, but the observations at hand do not allow us to confirm or reject this hypothesis. However, as discussed above, the explanation could also be that at least KIC 11197517 and KIC 11241343 have evolved off the main-sequence, based on the updated surface gravities from Huber *et al*.^59^

### The magnetic flux density

Notsu *et al*.^31^ calculated the magnetic flux <*fB*> using the residual flux in the core of the infrared Ca II line. Using the formulation in Schrijver *et al*.^27^, we have also calculated the magnetic flux using our measured S indices. Here, the Ca II H and K excess flux density 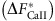, calculated as 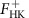 in the formulation by Mittag *et al*.^62^, is given as:


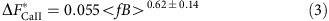


11 stars have been observed with both the Subaru and the LAMOST telescopes. Of these, one (KIC 11197517) has an S index so low that it results in a Ca II H and K flux density smaller than the basal flux and is therefore not used in the comparison shown in Supplementary Fig. 9. This comparison shows that, although the uncertainties are large on both types of estimates, the magnetic fluxes calculated from the Subaru observations generally seem to be larger than the magnetic fluxes calculated from LAMOST observations. The reason for this could be that the magnetic fluxes calculated based on the Subaru observations are not corrected for basal flux.

### Calculation of the flare rates

After we have calculated the S indices for the 5,648 main-sequence stars based on the LAMOST spectra with signal-to-noise ratios higher than 10 in the blue part of the spectrum, including the subset of 48 superflare stars, it is possible to calculate the flare rates. We calculate these flare rates for S indices of 0.169, 0.179 and 0.205 corresponding to the mean value of the Sun during solar cycle minimum, the mean value of the Sun during the whole solar cycle, and the mean value during solar cycle maximum^34^. The flare rates are given below, together with the associated uncertainties, assuming that the flare rates follow a Poisson distribution (Table 1).

We also performed the same calculation using only the stars with superflares having a total bolometric flare energy larger than 2·10^34^ erg (Table 2).

Similar flare rates were calculated by Maehara *et al*.^15^ and Shibayama *et al*.^16^ using rotation instead of chromospheric emission as the criterion for selecting Sun-like stars with magnetic activity levels similar to the Sun. This only works for active stars with large spots characterized by long lifetimes. For Sun-like stars with magnetic activity levels similar to the Sun, one should be very careful with such analysis. The reason is that Kepler would not be able to measure the rotation period of the Sun, as sunspots have lifetimes much shorter than the rotation period. We can therefore not assume that the stars that do not show rotational modulation in the Kepler observations rotate as slow or slower than the Sun. It could also be that the reason why we cannot see any rotational modulation in the Kepler observations is because the starspots of these stars have lifetime much shorter than their rotation periods.

### The significance of the flare fractions

To test the significance of the occurrence of flares, we performed a Monte Carlo simulation, where we randomly removed 48 stars from the 5,648 main-sequence stars and calculated the distribution of the S indices of these stars. This process was repeated 10,000,000 times and the resulting fractions were calculated as the mean values, and the uncertainty as the standard deviations (Table 3).

When these fractions are compared with the flare fractions, it is clear that the flare fractions are significantly different from the fractions obtained from randomly selecting 48 stars from the 5,648 main-sequence stars.

We also performed a bootstrapping test, where we compared the mean S index of the superflare stars with the mean S index of 48 stars randomly selected from the 5,648 stars. This process was also repeated 10,000,000 times. The mean S index of the superflare stars (0.2399) falls within the top 0.002% of the distribution of mean S indices based on 48 stars randomly selected from the 5,648 main-sequence stars (Supplementary Fig. 10).

### Calculation of the flare frequency

The frequency of solar flares in Fig. 7 (that is, the power-law distribution) is obtained from Fig. 6 in Crosby, *et al*.^64^ By noting that the power-law distribution in that figure passes through the point (10^31^ erg; 10^−32^ erg^−1^ day^−1^), we obtain the following equation for the power-law:


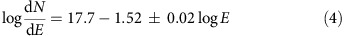


where *E* is measured in erg and d*N*/d*E* is measured in erg^−1^ year^−1^. The frequency of superflares is taken from Fig. 5b in Shibayama *et al*.^16^ Here, we have the exact values of the fit (Shibayama, T., personnel communication):


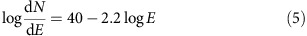


In order to include the contributions from stellar activity in this equation, we construct a new power-law relationship that should be a straight line in the log–log plot, going through a point at 10^33^ and one at 2·10^34^ erg. Using the values from Shibayama *et al*.^16^, these two points should be: (10^33^ erg; 10^−32.6^ erg^−1^ year^−1^) and (10^34.30^ erg; 10^−35.5^ erg^−1^ year^−1^). We then calculate the new points by taking 25% of the value at 10^33^ erg and 8% of the value at 2·10^34^ erg (these values are found above). In this way, we get (10^33^ erg; 10^−33.20^ ergs^−1^ year^−1^) and (10^34.30^ erg; 10^−36.6^ erg^−1^ year^−1^). The straight line that passes through these two points in a log–log plot, can be described by the following equation:


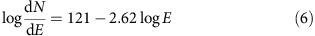


This relation is an upper limit (therefore, the arrow in Fig. 7), as we only calculate it for stars less active than the Sun at solar cycle maximum (0.205). It would arguably have been better to calculate the upper limit for stars less active than the mean activity level of the Sun over a solar cycle (0.179). However, the problem is that we do not have any stars hosting large superflares with total bolometric energies larger than 2·10^34^ erg, which have magnetic activity levels less than 0.179.

The slope of −2.62 can be compared with the slope of −2.0 found for slowly rotating stars by Shibayama *et al*.^16^ It is clear that the smaller slope obtained from the chromospheric emission measurements agrees better with the idea from the geological archives about a break, or roll-over, in the power-law distribution. The discrepancy between the two slopes suggests that slowly rotating stars can have large chromospheric emissions and likely large spots too. In other words, the chromospheric emission measurements suggest that it is less likely for the Sun to have a superflare compared with what is estimated based on the rotation measurements. When we use the slope of −2.62 to estimate the likelihood of the Sun hosting a superflare, we therefore implicitly assume that the Sun's chromospheric emission does not change dramatically. If the Sun, on the other hand, is capable of producing the large (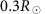) spot suggested by Shibata *et al*.^13^, then this assumption is likely violated, as the chromospheric emission of the Sun, in connection with such a large spot, would be dramatically larger than what we have observed so far.

### Contamination of the Kepler photometry

Each pixel on the Kepler photometer spans four times 4 arcs on the sky and each star is observed with an optimal aperture that consists of several pixels^65^. There is, therefore, a significant risk that multiple stars will be located in a given optimal aperture and thus that a given star is contaminated by a number of background stars. This again leaves the possibility that the observed superflares do not originate from the stars we assigned them to, but from background stars. This problem was discussed by Balona^66^, who used an analysis of the total bolometric flare energy to conclude that it is unlikely that the observation of superflares on A-type stars can be attributed to contamination. Notsu *et al*.^31^, on the other hand, obtained high-resolution spectra of 50 superflare stars with the Subaru telescope. These observations revealed that 16 out of these 50 superflare stars show signs of binarity. These signs were either radial velocity variations (1 star), Hα line profile variability (2 stars), double-lined profiles (9 stars) or visual binarity seen in the slit viewer images (4 stars).

Of the 48 superflare stars analysed in this study, 6 are identified as binaries by Notsu *et al*.^31^ These are: KIC 4045215, KIC 5445334, KIC 8226464, KIC 9653110, KIC 9764192 and KIC 11073910. In general, these stars all have S indices higher than the average for superflare stars. This is in agreement with the idea that binarity, through tidal coupling, can lead to increased magnetic activity^67^.

We have also examined the Hα line of the 48 superflare stars in our study to search for any signs of binarity. All 48 stars show nice narrow Hα absorption lines, with no indication of binarity. Owing to the low resolution of the LAMOST spectra, this does not rule out the possibility that more than six of these stars are binaries. It does, however, show that none of the 48 superflare stars are T Tauri stars. T Tauri stars are pre-main-sequence stars that show strong activity, especially in the Hα line and are known to host large flares^69^.

In order to test if our conclusions are affected by contamination of the Kepler photometry, we have repeated the whole analysis excluding the six binary stars. This did not change any of the conclusions presented in this study. The distribution of the measured activity in the now 42 superflare star ensemble is still significantly different from the distribution of the measured activity levels of all the 5,648 solar-like stars at a 6*σ* significance level. 36 out of 42 superflare stars have activity levels larger than 0.169 and 30 out of 42 have activity levels larger than 0.205. If we only look at superflares with total energies larger than 2·10^34^ erg, we observe that 26 out of 26 stars have activity levels larger than 0.169, whereas 22 out of 26 have activity levels larger than 0.205. We thus conclude that the conclusions presented here are not affected by contamination of the Kepler photometry.

## Additional information

**How to cite this article:** Karoff, C. *et al*. Observational evidence for enhanced magnetic activity of superflare stars. *Nat. Commun.* 7:11058 doi: 10.1038/ncomms11058 (2016).

## Supplementary Material

Supplementary Information Supplementary Figures 1-10 and Supplementary Table 1.

Supplementary Data 1 S index.

## Figures and Tables

**Table 1:** Fraction of superflare stars as a function of chromospheric emission.

S index smaller than	Fraction
0.169	13±5%
0.179	13±5%
0.205	25±7%

**Table 2:** The same as Table 1, but only bolometric flare energies larger than 2·10^34^ erg.

S index smaller than	Fraction
0.169	0%
0.179	0%
0.205	8±4%

**Table 3:** Mean fraction of random stars as a function of chromospheric emission.

S index smaller than	Fraction
0.169	17.5134%±0.0005%
0.179	29.6214%±0.0007%
0.205	63.1812%±0.0007%
