# Supplementary material for: Observational evidence for enhanced magnetic activity of superflare stars
Source: Nat Commun. 2016 Mar 24;7:11058. doi: 10.1038/ncomms11058 (PMC4820840; doi:10.1038/ncomms11058)
Supplement: Supplementary Information — Supplementary Figures 1-10 and Supplementary Table 1. [file ncomms11058-s1.pdf]

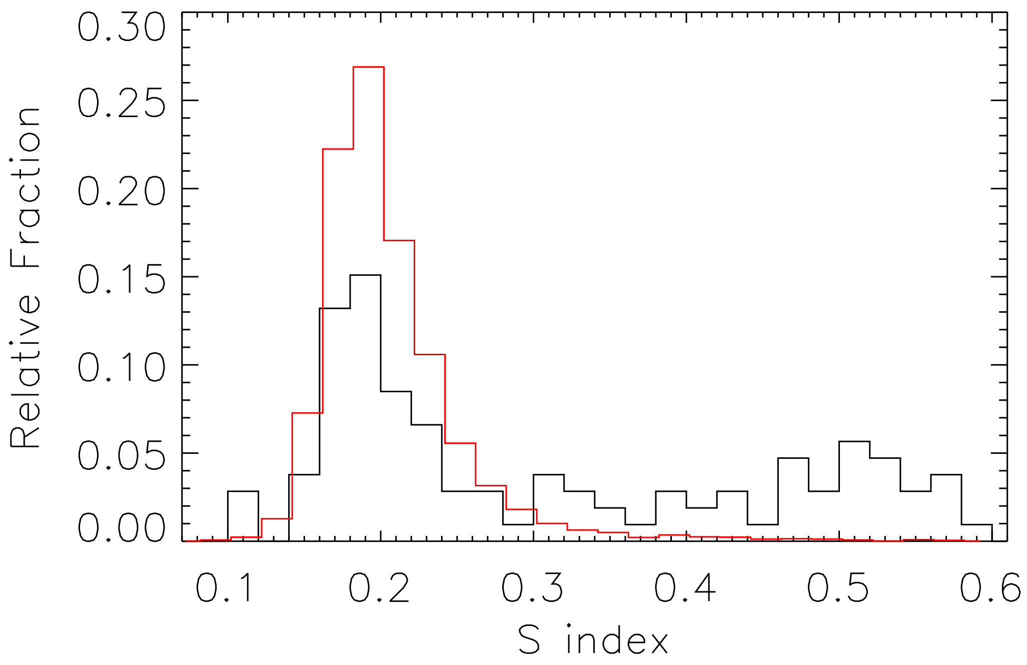

Supplementary Figure 1 Comparison of the distribution of S indices measured by LAMOST (red) and the distribution measured by Isaacson & Fischer[57] (black). Isaacson and Fischer observe more high-activity stars with S index higher than 0.4 than we observe based on LAMOST. The distributions are, however, identical for S indices between 0.1 and 0.3. This is confirmed by a Kolmogorov-Smirnov test, which shows that the two distributions are identical between 0.1 and 0.3 with a 92.91% significance level.

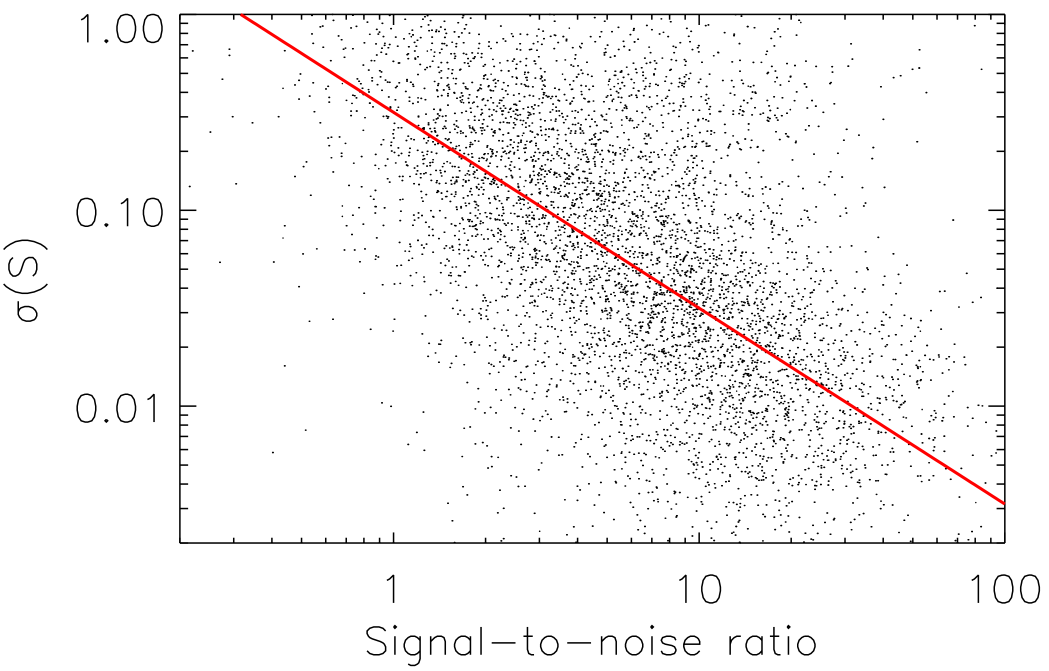

Supplementary Figure 2 Relation between the standard deviation of different measurements of the S indices for the same stars in different LAMOST fields and the signal-to-noise ratio in the blue part of the spectra. A log-log relation is seen and the red line represents the fit used for estimating the uncertainty of the measurements of the S indices.

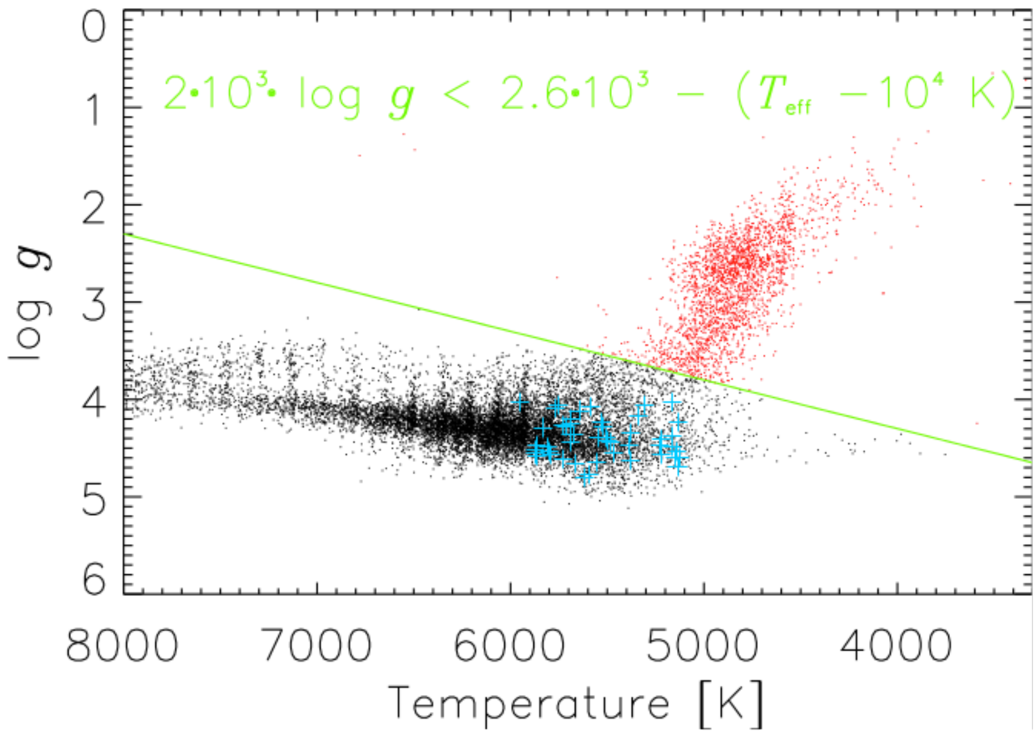

Supplementary Figure 3 Hertzsprung-Russell diagram for the *Kepler* stars observed with LAMOST using input values from KIC. The effective temperature and the surface gravity are from Brown et al.[59]. The evolved stars are marked with red and the superflare stars are marked with blue. Main-sequence stars are defined as all stars below the green line, which is given by the equations on the figure.

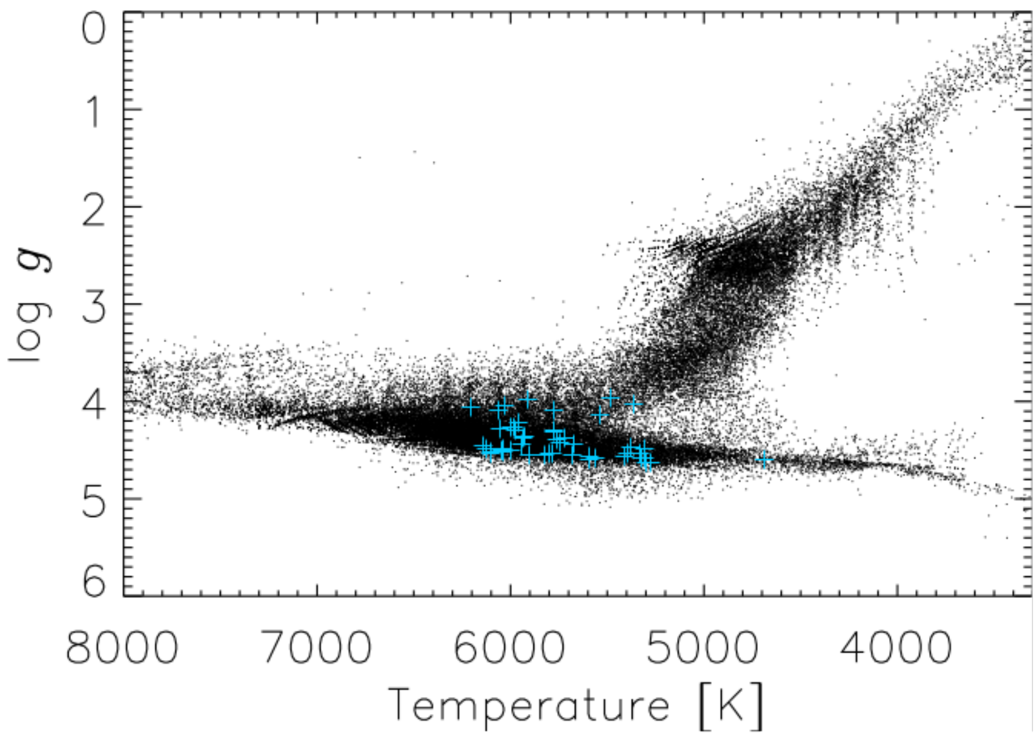

Supplementary Figure 4 Hertzsprung-Russell diagram for the *Kepler* stars observed with LAMOST using input values from Huber et al. The same as Fig. 4, but this time the effective temperature and the surface gravity comes from Huber et al.[60].

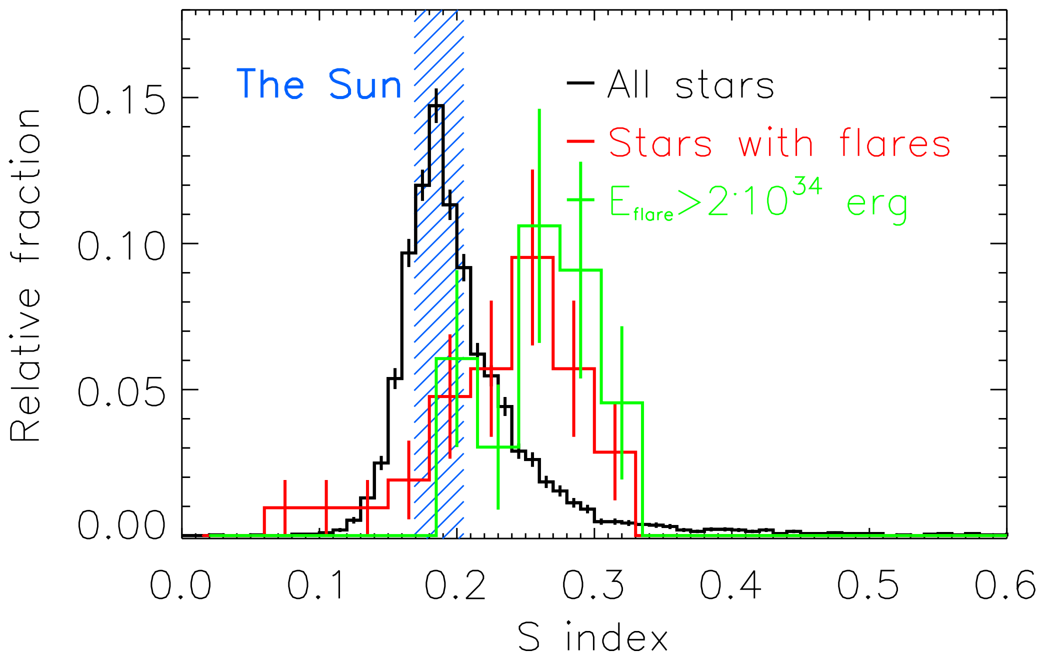

Supplementary Figure 5 Histogram of the activity distribution of solar-like stars using input values from Huber et al. The same as Fig. 2, but this time the effective temperature and the surface gravity comes from Huber et al.[60], which means that only 35 of the 48 superflare stars now fall in the 5100 to 6000 K temperature range.

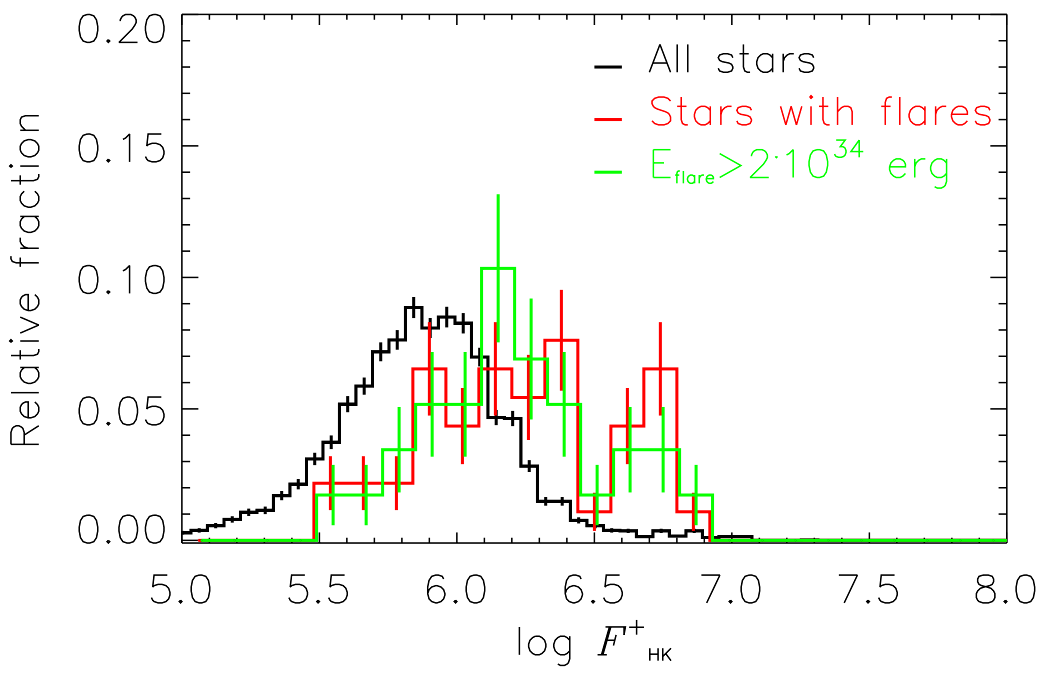

Supplementary Figure 6 Histogram of the chromospheric excess flux F^{+}_{HK}. The figure shows the same as Fig. 2, but here fore the chromospheric excess flux instead of the S index. The chromospheric excess flux is calculated using the S indices and the formulation in Mittag et al.[63] As for the S index, it is seen that distribution of the superflare stars is clearly different form the distribution of the Sun-like stars.

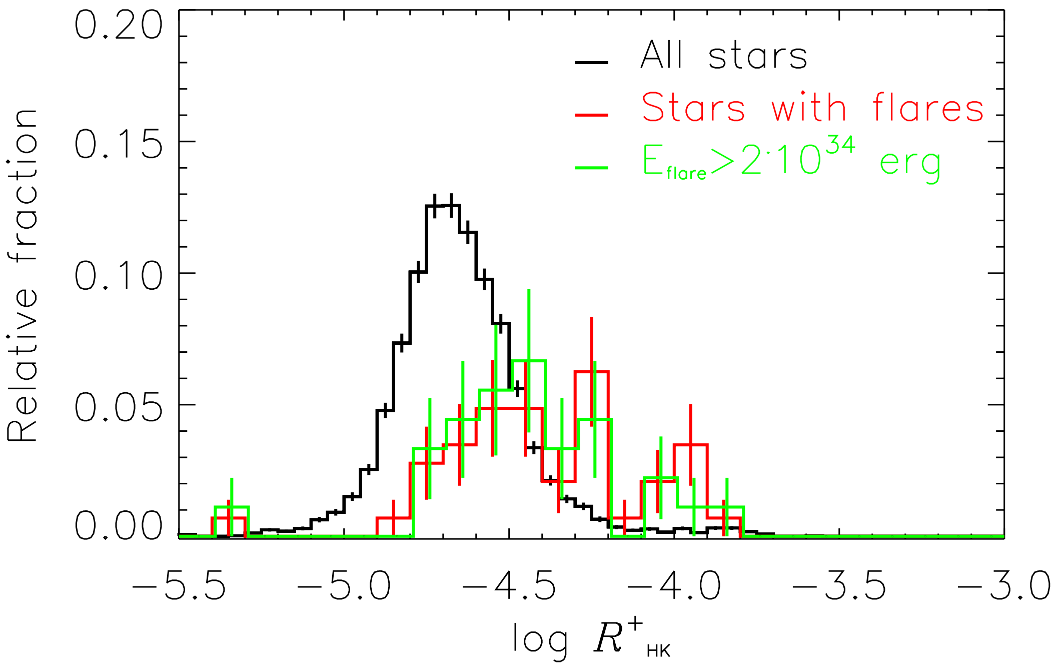

Supplementary Figure 7 Histogram of the flux-related quantity R^{+}_{HK}. The figure shows the same as Fig. 2, but here for the R^{+}_{HK} instead of the S index. R^{+}_{HK} is calculated using the S indices and the formulation in Mittag et al.[63] Again, it is seen that distribution of the superflare stars is clearly different form the distribution of the Sun-like stars.

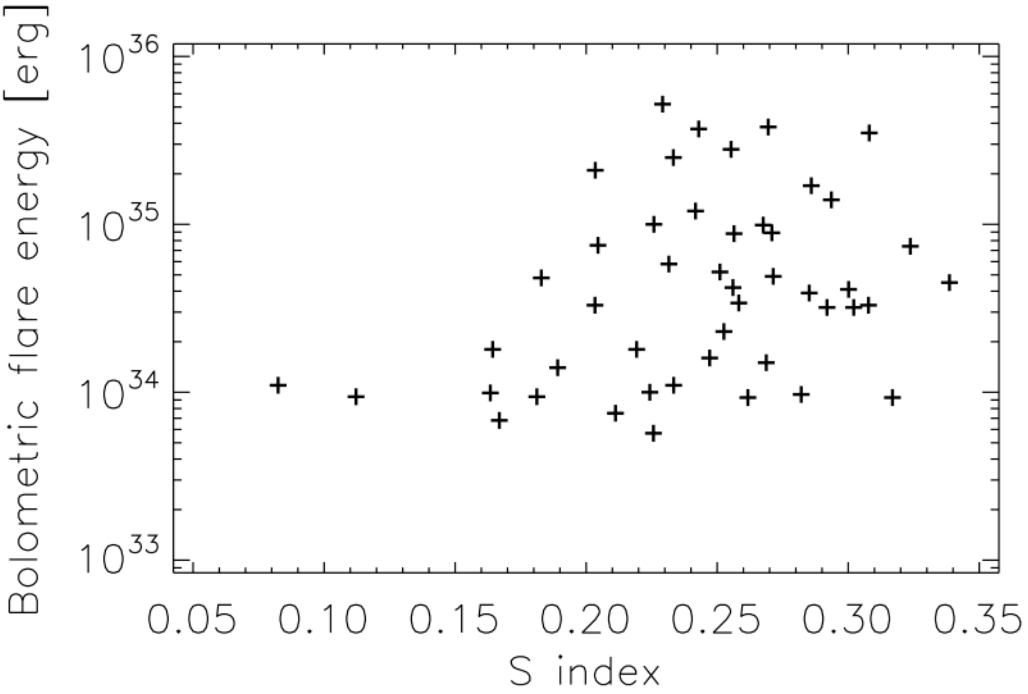

Supplementary Figure 8 Relationship between the flare energy and activity. The total bolometric flare energy is calculated by Shibayama et al.[16]. Though no clear relation can be seen, it is clear that no stars with low activity levels have been observed to produce large superflares.

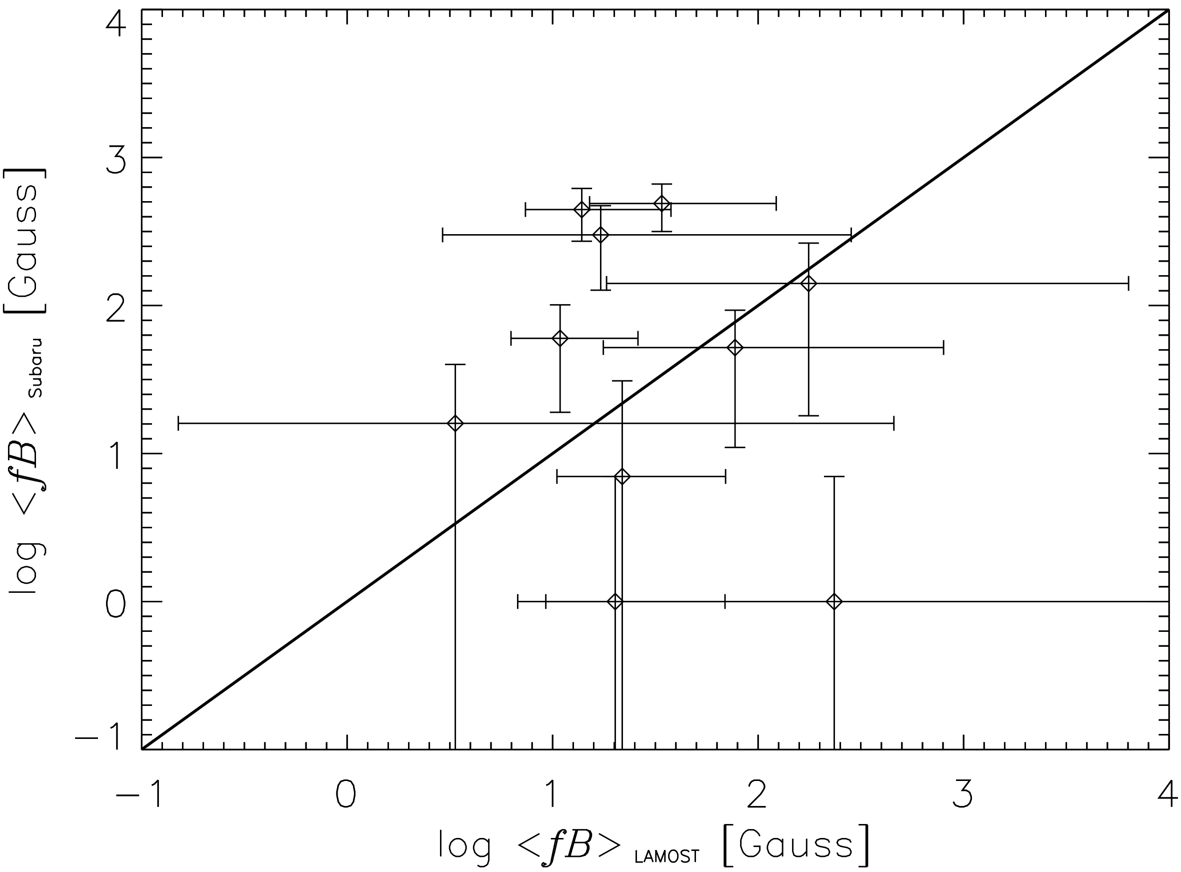

Supplementary Figure 9 The magnetic flux density. The figure compares the magnetic flux density of 10 stars measured with both the Subaru and the LAMOST telescopes. The solid line shows a 1:1 relationship. Note that we only plot 10 stars here as the S index of KIC 11197517 is so low that is gets a negative chromospheric flux using the formulation by Mittag et al.[63]. The error bars are calculated from the uncertainty on the chromospheric flux and the uncertainty on the exponent in equation 3.

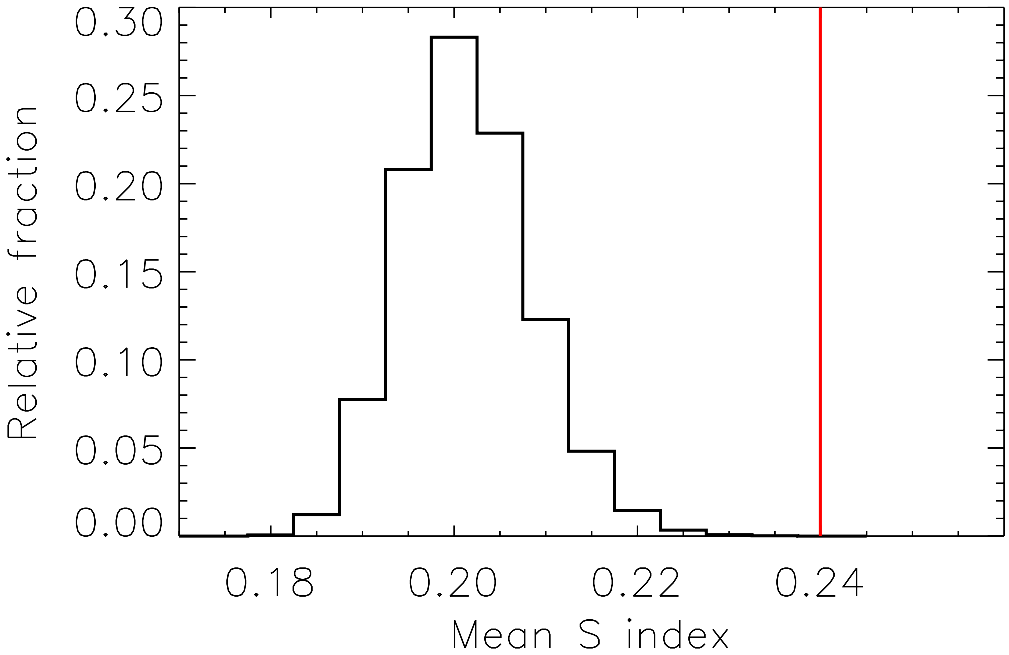

Supplementary Figure 10 The mean S index. The mean S index of 48 stars randomly selected from the 5648 main-sequence stars. The mean value of the S index of all Sun-like stars is 0.197, whereas the mean value of the superflare stars is 0.2399, which shows that the observed value for the superflare stars (red line) falls with in the top 0.002% of solar-like stars in general.

ID	$T_{\rm eff}$	log g	S index
KIC 2850378	5595	4.77	0.11
KIC 2862041	5504	4.39	0.18
KIC 3217974	5221	4.57	0.21
KIC 4045215	5229	4.47	0.22
KIC 4354963	5388	4.47	0.16
KIC 4633721	5165	4.49	0.17
KIC 4646848	5953	4.03	0.26
KIC 5281818	5525	4.28	0.25
KIC 5445334	5137	4.69	0.25
KIC 5522535	5732	4.26	0.32
KIC 5616432	5672	4.35	0.26
KIC 5729515	5529	4.23	0.29
KIC 5953596	5161	4.37	0.29
KIC 6503434	5714	4.29	0.20
KIC 6544160	5692	4.20	0.20
KIC 6633742	5807	4.49	0.18
KIC 6865484	5688	4.36	0.26
KIC 7017862	5789	4.57	0.24
KIC 7667812	5830	4.29	0.23
KIC 8091757	5871	4.56	0.34
KIC 8226464	5754	4.05	0.24
KIC 8479655	5126	4.60	0.27
KIC 8564441	5798	4.53	0.23
KIC 8621939	5688	4.44	0.27
KIC 8848528	5586	4.08	0.20
KIC 9152469	5864	4.57	0.28
KIC 9652680	5618	4.80	0.30
KIC 9653110	5223	4.41	0.33
KIC 9706078	5692	4.26	0.19
KIC 9764192	5551	4.64	0.30
KIC 9944137	5725	4.62	0.16
KIC 10346286	5544	4.39	0.23
KIC 10352333	5380	4.35	0.26
KIC 10471412	5771	4.08	0.23
KIC 10528093	5143	4.53	0.29
KIC 10593098	5129	4.23	0.27
KIC 10646889	5483	4.44	0.26
KIC 10796663	5336	4.16	0.23
KIC 10921242	5868	4.51	0.31
KIC 11073910	5381	4.64	0.29
KIC 11140181	5463	4.55	0.27
KIC 11197517	5162	4.02	0.08
KIC 11241343	5305	4.06	0.12
KIC 11303472	5150	4.60	0.23
KIC 11455711	5664	4.67	0.22
KIC 11610797	5865	4.46	0.30
KIC 11972298	5498	4.43	0.25
KIC 12109550	5645	4.11	0.27

Supplementary Table 1. S indices and stellar parameters for 48 superflare stars
